# DNA G-Quadruplex in NRP1 Promoter Facilitates SARS-CoV-2 Infection

**DOI:** 10.3390/ijms25084422

**Published:** 2024-04-17

**Authors:** Pihai Gong, Rongxin Zhang, Ke Xiao, Huiling Shu, Xinxiu Li, Hong Fan, Xiao Sun

**Affiliations:** 1 State Key Laboratory of Digital Medical Engineering, School of Biological Science and Medical Engineering, Southeast University, Nanjing 211189, China; 2 Department of Medical Genetics and Developmental Biology, School of Medicine, Southeast University, 87 Dingjiaqiao Road, Nanjing 210009, China; dbxl2019@163.com

**Keywords:** G-quadruplexes, SARS-CoV-2, gene regulation, COVID-19

## Abstract

Severe acute respiratory syndrome coronavirus 2 (SARS-CoV-2) infection continues to raise concerns worldwide. Numerous host factors involved in SARS-CoV-2 infection have been identified, but the regulatory mechanisms of these host factor remain unclear. Here, we report the role of G-quadruplexes (G4s) located in the host factor promoter region in SARS-CoV-2 infection. Using bioinformatics, biochemical, and biological assays, we provide evidence for the presence of G4 structures in the promoter regions of SARS-CoV-2 host factors NRP1. Specifically, we focus on two representative G4s in the *NRP1* promoter and highlight its importance in SARS-CoV-2 pathogenesis. The presence of the G4 structure greatly increases NRP1 expression, facilitating SARS-CoV-2 entry into cells. Utilizing published single-cell RNA sequencing data obtained from simulated SARS-CoV-2 infection in human bronchial epithelial cells (HBECs), we found that ciliated cells with high levels of *NRP1* are prominently targeted by the virus during infection. Furthermore, our study identifies E2F1 act as a transcription factor that binds to G4s. These findings uncover a previously unknown mechanism underlying SARS-CoV-2 infection and suggest that targeting G4 structures could be a potential strategy for COVID-19 prevention and treatment.

## 1. Introduction

The global pandemic of coronavirus disease 2019 (COVID-19), caused by the novel SARS-CoV-2, has rapidly propagated and led to extensive disruptions in health, society, and the economy. The SARS-CoV-2 virus not only causes severe respiratory disease but also is implicated in various other health issues such as cardiovascular, hepatic and renal complications [1]. Moreover, the ongoing evolution and increased transmissibility of SARS-CoV-2 variants pose challenges to the development of effective therapeutic strategies [2,3]. Despite significant progress made by the global scientific community in the past, including the development and clinical trials of vaccines and the implementation of preventive measures to control viral spread, there is still a lack of specific and effective drugs for the treatment of SARS-CoV-2 infection. Therefore, a comprehensive understanding of the pathological mechanisms and infection processes of SARS-CoV-2 is crucial in order to discover new antiviral treatment approaches and drug targets.

In addition to ACE2 and TMPRSS2, an increasing number of host factors are proving essential in SARS-CoV-2 infection. Neuropilin-1 (NRP1) is a transmembrane receptor protein that plays a critical role in various physiological and pathological processes [4]. It was initially discovered in the nervous system and plays a crucial role in neural development and angiogenesis [5,6]. Recent studies have indicated that NRP1 functions as an additional receptor, working in conjunction with ACE2 and contributing to the process of virus entry. Specifically, NRP1 interacts with the S protein of SARS-CoV-2 and facilitates the binding between the virus and host cells [7,8]. Motivated by NRP1 pathological importance, specific inhibitors targeting NRP1 are being developed to combat the COVID-19 pandemic. However, the mechanisms underlying the regulation of *NRP1* in COVID-19 etiology remain unknown.

G-quadruplexes are non-classical secondary structures formed in DNA or RNA sequences and are involved in regulating various biological processes [9]. Studies on multiple viruses have identified G4s as potential antiviral targets, such as Hepatitis C virus (HCV), Zika virus (ZIKV), and Ebola virus (EBOV) [10]. The RNA G-quadruplex (RNA G4) in *TMPRSS2* reduces SARS-CoV-2 infection, which is the first discovery of its impact on the SARS-CoV-2 host factor [11]. Researchers have uncovered the role of RNA G4 in SARS-CoV-2 infection mechanisms and spotlighted RNA G4 as a potential target for COVID-19 prevention and treatment. Whether the G4s in the promoter regions of host factors associated with SARS-CoV-2 entry can serve as therapeutic targets for COVID-19 has become a focus of research. Therefore, exploring the potential involvement of G4s in host factor promoters is crucial for the development of effective treatments for COVID-19.

G4 structures commonly found in gene promoter regions can impact transcription [12]. However, there is a significant research gap regarding how G4 structures in the promoter regions influence the invasion of SARS-CoV-2. Here, we aimed to elucidate the association between G4 structures in the promoter regions of *NRP1* and SARS-CoV-2 invasion. Firstly, we screen the promoter regions of multiple host factors and select a key host factor NRP1 for further detailed investigation. Subsequently, using native gel electrophoresis, circular dichroism (CD), and ChIP-PCR, we confirm the stable presence of G4 structures in the promoter region of *NRP1*. Furthermore, we explore the effects of G4 structures on the transcriptional regulation of *NRP1* and assess the impact of G4 structure disruption on SARS-CoV-2 invasion through functional experiments. Lastly, as G4 structures serve as important hubs for transcription factor binding, we investigate the specific transcription factors that bind to G4 structures in the host factor’s promoter region using techniques like DNA pull-down and ChIP-PCR. Our study may provide important insights for the development of novel antiviral strategies.

## 2. Results

### 2.1. Identification of Two G-Quadruplex Structures in the NRP1 Promoter

**Figure 1:**
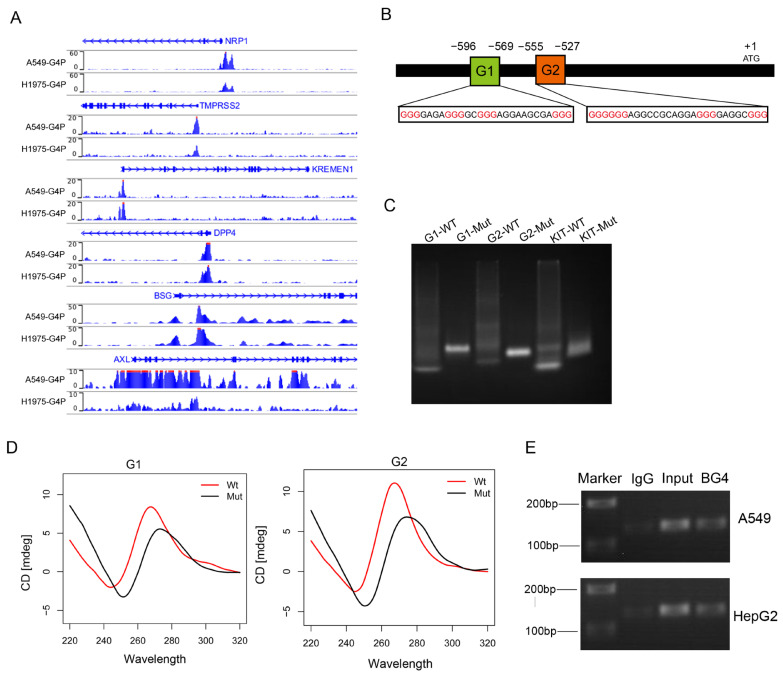
The *NRP1* promoter contains two G-quadruplex structures. (**A**) G4 signals in the promoters of genes *NRP1*, *TMPRSS2*, *KREMEN1*, *DPP4*, *BSG*, and *AXL* visualized using the IGV browser. (**B**) Promoter structure of the human *NRP1* gene and the location of G4s on the chromosome. Consecutive guanine residues are highlighted in red. (**C**) Non-denaturing gel electrophoresis experiment showing the electrophoretic mobility of wild-type and mutant sequences of the two G4s in the promoter. The G4 sequence in the promoter region of the *KIT* gene serves as a positive control. (**D**) CD spectroscopy analysis of wild-type and mutant sequences of the two G4s. (**E**) Detection of the two G-quadruplex structures using ChIP technology combined with PCR.

Previous studies have identified several proteins involved in SARS-CoV-2 entry into host cells, including ACE2, NRP1, TMPRSS2, KREMEN1 [13], DPP4 [14], BSG [15], and AXL [16]. To investigate whether the expression of these receptors is regulated by G-quadruplexes and consequently affects the invasion of the SARS-CoV-2, we downloaded the G4 ChIP-seq data from the GEO database for the A549 and H1975 cell lines [17]. We observed G4 ChIP-seq signals in the promoters of *NRP1*, *TMPRSS2*, *KREMEN1*, *DPP4*, *BSG*, and *AXL* (Figure 1A). We focused on G4s in the promoter of *NRP1*, which is a common cellular entry determinant for SARS-CoV-2. First, we obtained the promoter sequence of NRP1 and predicted the presence of potential G-quadruplex sequences (PQSs) in the promoter (Figure 1B). Putative G4-forming sequences were predicted using the QGRS-mapper algorithm [18]. We selected the top two highest-scoring canonical G4s and found that these G4s were also located at the transcription start site (TSS), which is consistent with the results from G4 ChIP-seq analysis. This result indicates that two G4s may form secondary structures in the promoter of *NRP1*. 

To validate whether these potential sequences can fold into secondary structures, we synthesized wild-type (WT) and G4-mutant (Mut) DNA sequences and performed non-denaturing gel electrophoresis experiments. Under the K^+^ condition, we observed that WT migrated faster than Mut, suggesting that WT folded into a compact secondary structure. The results indicated that both G4 sequences could fold into G4 structures (Figure 1C). In addition, the top two PQSs were chosen for experimental verification by using circular dichroism (CD); CD spectra experiments confirmed the presence of the two G4 structures (Figure 1D). Finally, we employed ChIP-seq technology combined with PCR to detect the formation of these two G4 structures in A549 and HepG2; these data demonstrated that G4 sequences were enriched in the *NRP1* promoter, and these results confirmed the presence of G4 structures in vivo (Figure 1E). These consistent results indicate the presence of two canonical G4 structures in the *NRP1* promoter, suggesting their potential crucial role in the transcriptional regulation of *NRP1*.

### 2.2. The Expression Level of NRP1 Is Upregulated in Cells Infected with SARS-CoV-2

**Figure 2:**
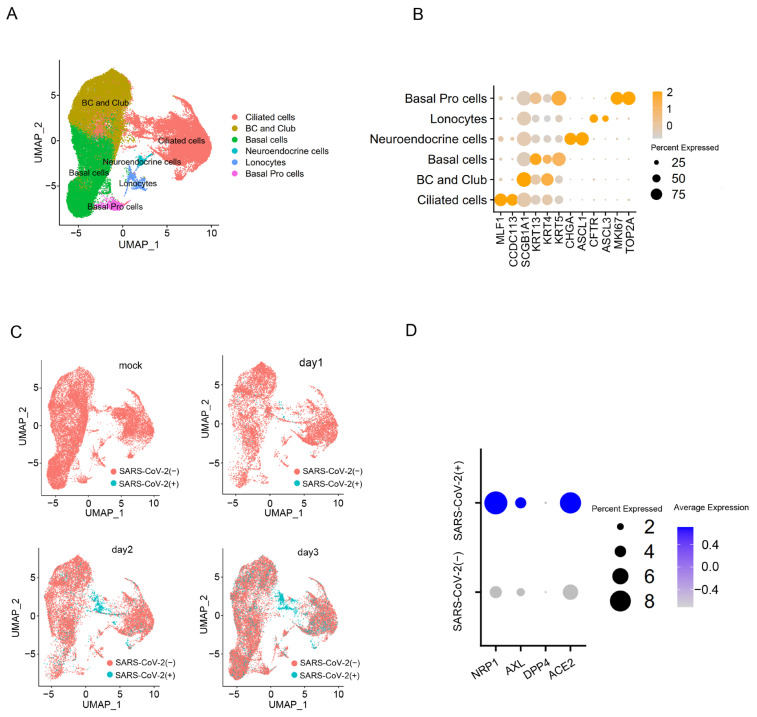
SARS-CoV-2 is prone to infecting ciliated cells with a high expression level of *NRP1*. (**A**) Visualize manually annotated cell clusters using UMAP. (**B**) Dot plot displaying gene expression patterns of cluster-enriched markers. (**C**) UMAP visualization on SARS-CoV-2-infected cells in batch-corrected HBEC samples. (**D**) Compare the expression levels of genes *NRP1*, *AXL*, *DPP4*, and *ACE2* in ciliated cells between infected and uninfected cells with SARS-CoV-2.

In order to assess the expression level of *NRP1* in cells infected with the SARS-CoV-2, a published single-cell RNA sequencing dataset was analyzed using the Seurat package. This dataset was from human bronchial epithelial cells (HBECs) with or without SARS-CoV-2 infection [19]. After quality control, we obtained 62,747 cells and subjected them to clustering analysis. We identified six major clusters representing classic epithelial cell types, including Ciliated cells, Basal cells, BC and Club cells, Neuroendocrine cells, Lonocytes and Basal Pro cells (Figure 2A). Differential gene expression analysis (DEG analysis) unveiled the expression of classical epithelial cell type-specific markers within cell clusters (Figure 2B). Mapping virus-infected cells in specific epithelial cell types reveals that ciliated cells are the main target of SARS-CoV-2 infection, and their proportion increases with the duration of SARS-CoV-2 virus infection (Figure 2C). Within the ciliated cells, we found that the expression levels of *NRP1*, *ACE2* and *AXL* were significantly higher in cells infected with SARS-CoV-2 compared to uninfected cells (Figure 2D). These results suggest that the expression levels of the host factor may determine the extent of viral invasion. To investigate the impact of G4s on these receptors and their role in SARS-CoV-2 infection, we focused our study on *NRP1* for further research.

### 2.3. The G-Quadruplex Structures in the Promoter Increase the Expression Levels of NRP1

**Figure 3:**
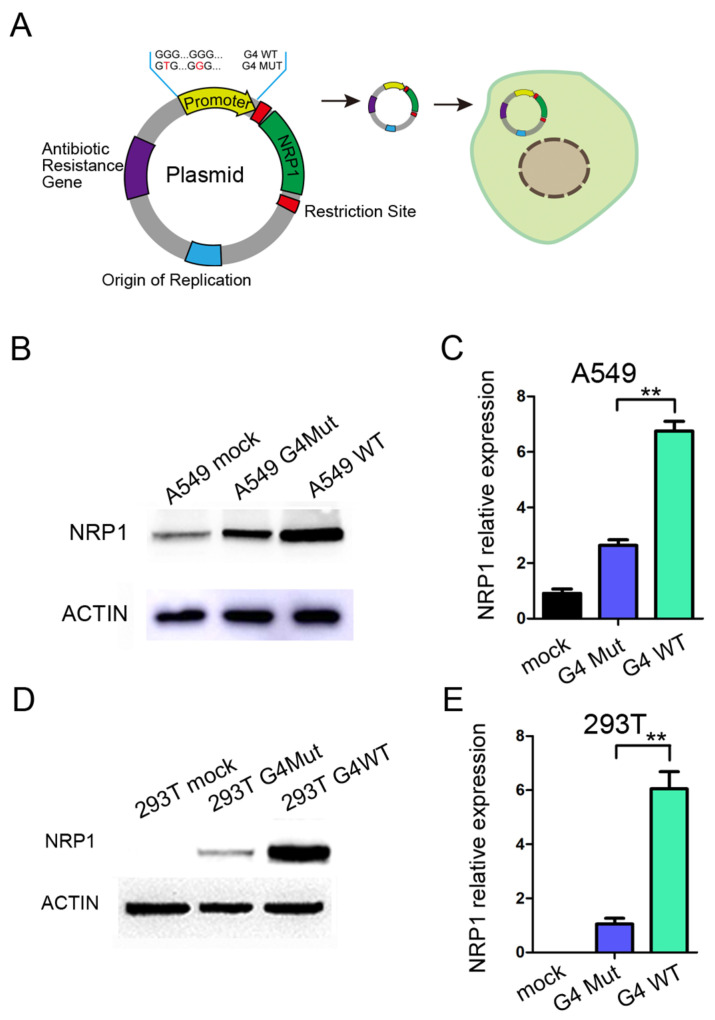
The impact of G4 structure on NRP1 expression. (**A**) Schematic diagram of *NRP1* overexpression vector and modifications to the promoter. (**B**) Overexpression of wild-type and mutant G4 with *NRP1* full-length vectors in A549 cell line, followed by Western blot analysis to assess the impact of G4 structures on NRP1 expression. (**C**) Overexpression of wild-type and mutant G4 with *NRP1* full-length vectors in A549 cell line, followed by qPCR analysis to evaluate the influence of G4 structures on *NRP1* expression. All tests were performed at least three times. Data were expressed as mean ± SEM. ** *p* < 0.01. (**D**) Overexpression of wild-type and mutant G4 with *NRP1* full-length vectors in HEK293T cell line, followed by Western blot analysis to investigate the effect of G4 structures on NRP1 expression. (**E**) Overexpression of wild-type and mutant G4 with *NRP1* full-length vectors in HEK293T cell line, followed by qPCR analysis to examine the impact of G4 structures on *NRP1* expression. All tests were performed at least three times. Data were expressed as mean ± SEM. ** *p* < 0.01.

To further investigate the role of G4s located in the promoter of *NRP1* during the transcription process, we constructed a fused vector with the full-length sequence of *NRP1*, while the promoter region of the vector was replaced with a 200 bp sequence containing the G4s of *NRP1* promoter regions (Figure 3A). Additionally, we introduced a mutant version of the promoter sequence with both two G4s mutated. This mutation should prevent the formation of G4 by a synonymous substitution. We then transfected the vector into A549 and HEK293T cells. A549 cells endogenously express *NRP1*, and after transfection with the vector, we observed a significant increase in *NRP1* expression compared to the mutant control, which was consistent with both the mRNA and protein levels (Figure 3B,C). Furthermore, in HEK293T cells that do not endogenously express NRP1, we found that transfection with the vector led to substantial NRP1 overexpression at both the mRNA and protein levels compared to the mutant control (Figure 3D,E). Collectively, these compelling findings support that the formation of G4 within the *NRP1* promoter exerts a pronounced transcriptional promoting role on *NRP1* expression. The presence of the G4 sequence within the promoter region not only promotes NRP1 expression in A549 cells but also initiates de novo NRP1 expression in HEK293T cells, suggesting that G4s in the *NRP1* promoter play a key role in promoting NRP1 expression.

### 2.4. Sequestration or Disruption of G4 Structures in the Promoter Inhibits the Expression Levels of NRP1

**Figure 4:**
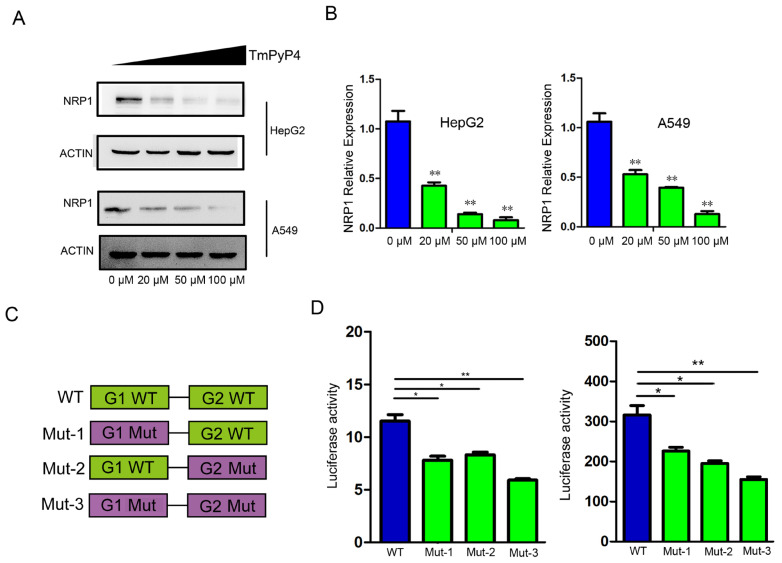
The impact of G4 sequestration or disruption on NRP1 expression. (**A**) TmPyP4 treatment of cell lines A549 and HepG2, Western blot analysis of NRP1 expression levels. (**B**) TmPyP4 treatment of cell lines A549 and HepG2, qPCR analysis of *NRP1* expression levels. All tests were performed at least three times. Data were expressed as mean ± SEM. ** *p* < 0.01. (**C**) Schematic diagram of the design of wild-type and mutant constructs on a luciferase activity reporter vector. (**D**) Luciferase promoter assay performed in cell lines. Fluorescent luciferase activity was measured using a dual luciferase reporter gene system. Results are presented as the average of two independent experiments with three replicates each. The bar graph represents the mean ± standard deviation. * *p* < 0.05, ** *p* < 0.01.

In order to determine the impact of G4 on NRP1 expression, we designed two experiments to investigate the impact of G4 structures elimination on NRP1 expression. We first treated HepG2 and A549 cells with G4-specific ligands TmPyP4, which hindered the interaction between G-quadruplexes and proteins [20], and measured the protein levels of NRP1. Compared to the control group (0 μM), we noted a substantial reduction in NRP1 protein levels in cells treated with TmPyP4 in a dose-dependent manner (Figure 4A). After treatment with different dose of TMPyP4, mRNA was extracted and analyzed by qRT-PCR. We observed a significant decrease in the mRNA expression levels of *NRP1* compared to the control group, which was consistent with protein detection results (Figure 4B). To further demonstrate that the disruption of G4 structures affects the expression levels of NRP1, we constructed wild and mutant-type luciferase reporter gene vectors (Figure 4C). In A549 and HepG2 cells, which endogenously express NRP1, the luciferase activity of the mutant vectors was significantly reduced compared to that of the wild-type vectors (Figure 4D). These findings indicate that the disruption of G4s in the *NRP1* promoter influences *NRP1* transcription. G4s sequestration or disruption is expected to hinder the binding of transcription factors to the promoter region, thereby impacting the regulation of *NRP1* expression.

### 2.5. G4s Affect the Invasion of SARS-CoV-2 by Regulating the Expression of NRP1

**Figure 5:**
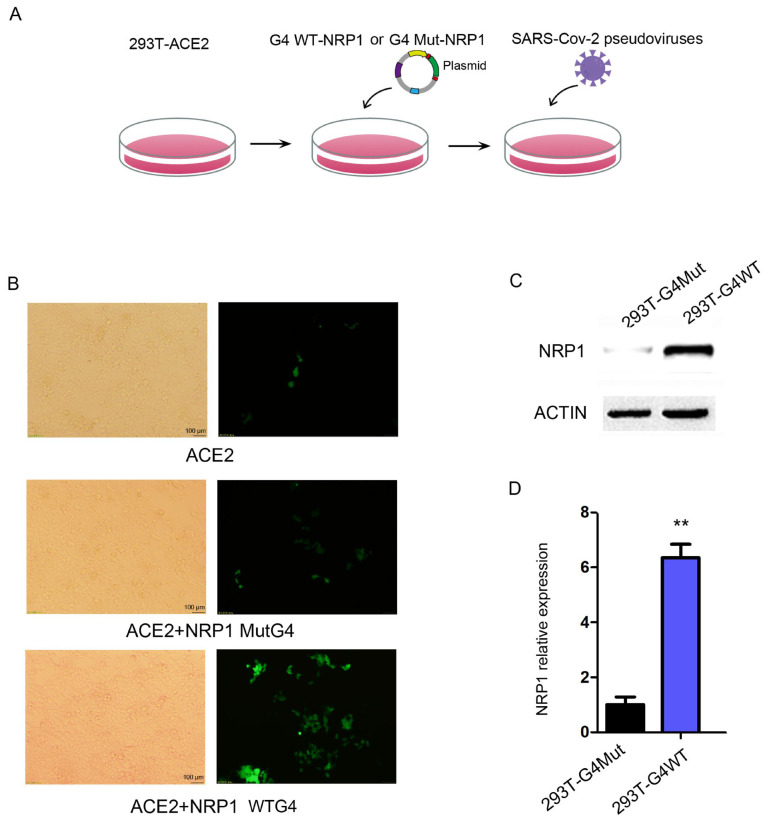
G4 facilitates SARS-CoV-2 invasion by regulating NRP1 expression. (**A**) Flowchart of the pseudovirus infection experiment. (**B**) Transfection of wild-type G4 and mutant G4 promoter-modified *NRP1* full-length vectors into *ACE2*-transfected 293T cells to study the impact of G4 on SARS-CoV-2 invasion. (**C**) Western blot analysis to examine the effects of wild-type G4 and mutant G4 promoters on NRP1 expression in the pseudovirus infection experiment. (**D**) qPCR analysis to assess the effects of wild-type G4 and mutant G4 promoters on *NRP1* expression in the pseudovirus infection experiment. All tests were performed at least three times. Data were expressed as mean ± SEM. ** *p* < 0.01.

Based on the findings that G4s are located in the promoter region of *NRP1* and influence *NRP1* expression levels, we hypothesized that G4 structures in the *NRP1* promoter might regulate SARS-CoV-2 infection. To test this hypothesis, we employed a developed pseudovirus system to monitor the viral entry step, in which vesicular stomatitis virus (VSV) was pseudotyped with SARS-CoV-2 S glycoprotein. The pseudoviruses were then used to infect human cells, and they expressed a fluorescence protein for quantification. We cultured HEK293T cells and performed the following experimental procedure, as depicted in Figure 5A. Firstly, HEK293T cells stably expressed the ACE2 protein. Secondly, we transfect HEK293T with the aforementioned full-length *NRP1* vector containing the wild or mut-type G4 promoter. Lastly, we transfected the cells with pseudovirus particles expressing green fluorescent protein (GFP) to measure the extent of viral invasion on the fifth day. The pseudovirus entry efficiency was higher in the WT group compared to the control group (Figure 5B). Meanwhile, we collected RNA and protein from both the control and experimental groups, confirming significant changes in the protein and mRNA expression levels of *NRP1* during this simulated process (Figure 5C,D). These findings suggest that G4s can modulate *NRP1*, thereby facilitating the invasion of SARS-CoV-2.

### 2.6. G4 Recruit E2F1 in the Promoter Region, Regulating NRP1 Expression

**Figure 6:**
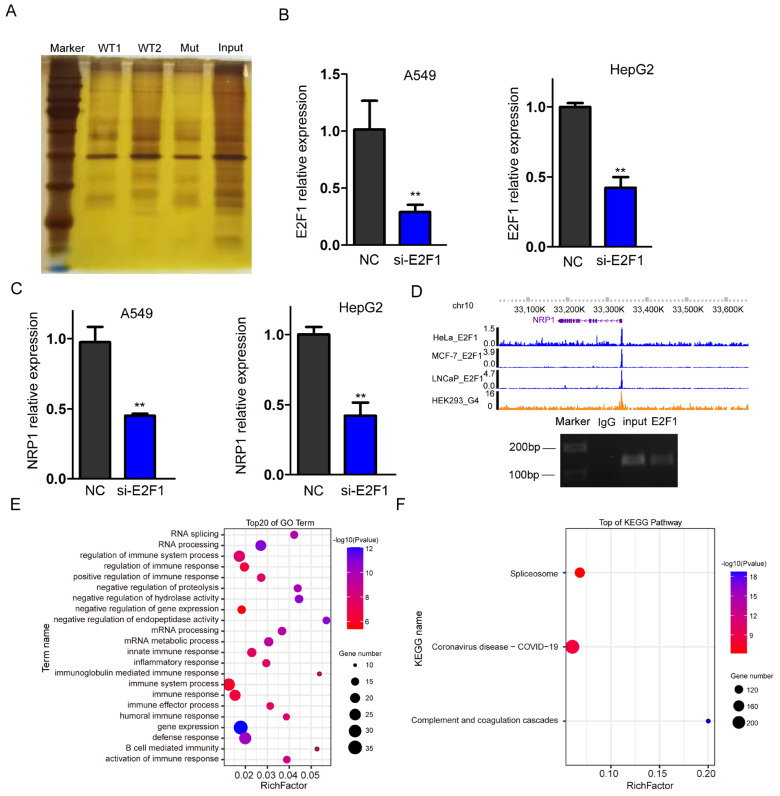
Transcription factor E2F1 binds to the *NRP1* promoter. (**A**) PAGE electrophoresis of proteins bound in DNA pull-down assay. (**B**) qPCR analysis to assess the efficiency of *E2F1* knockdown in A549 and HepG2 cell lines. All tests were performed at least three times. Data were expressed as mean ± SEM. ** *p* < 0.01. (**C**) qPCR analysis of *NRP1* expression levels following *E2F1* knockdown in A549 and HepG2 cell lines. All tests were performed at least three times. Data were expressed as mean ± SEM. ** *p* < 0.01. (**D**) IGV browser displaying the binding signal of E2F1 at the *NRP1* promoter (upper panel). ChIP-PCR assays showed that E2F1 binds to the G4s locus in *NRP1* promoter in A549 (lower panel). (**E**) GO analysis of all proteins captured in the DNA pull-down assay. (**F**) KEGG pathway analysis of all proteins captured in the DNA pull-down assay.

To identify the specific transcription factors recruited by G4 structures in the *NRP1* promoter, we synthesized promoter DNA sequences containing wild and mutant-type G4. After denaturing and annealing the biotin-labeled DNA, DNA pull-down assay was performed with a DNA probe containing the G4 structure and nuclear proteins isolated from A549 cells. Then, the pulled-down proteins were subjected to polyacrylamide gel electrophoresis (PAGE) and gel excision (Figure 6A). Finally, we used LC-MS/MS analysis to identify potential transcription factors or proteins that interact with the G4 structures. To select a target protein from the mass spectrometry data, various criteria were taken into account, including score, intensity, and the target protein’s role as a transcription factor. After selecting, we focused on validating the involvement of E2F1, which is the only transcription factor in the list (Supplementary Data). E2F1 is known to have DNA-binding motifs enriched in G-rich sequences, which have higher probability folding into the G-quadruplex. To validate the promotive role of E2F1 on NRP1 expression, small interfering RNA (siRNA) was used to knock down *E2F1* expression levels in A549 and HepG2 cells. qPCR analysis confirmed the successful reduction in *E2F1* expression (Figure 6B). Subsequently, we examined the expression levels of *NRP1* and observed a significant suppression (Figure 6C). These data indicate that E2F1 can influence the transcription of *NRP1*. To investigate whether E2F1 directly binds to G4s in the promoter, we downloaded ChIP-seq data from HeLa [21], MCF-7 [22], and LNCaP [23] cells and found an occupancy of E2F1 in the promoter of *NRP1* (Figure 6D upper panel). We also employed ChIP-PCR to confirm the binding of E2F1 at the G4s locus (Figure 6D lower panel). These data suggest that E2F1 is a transcription factor recruited by the G4 structure, which promotes *NRP1* expression. Furthermore, we performed GO analysis on all the interacting proteins with G4s, and the results showed a significant association with immune processes, which is consistent with the known function of *NRP1* as an immune-related molecule [24,25,26] (Figure 6E). KEGG analysis of all the proteins revealed involvement in pathways related to COVID-19, suggesting a potential relationship between G4 sequences in the *NRP1* promoter and COVID-19 (Figure 6F).

Together, these results suggest that G4s in the *NRP1* promoter can recruit E2F1 and effectively facilitate SARS-CoV-2 infection.

## 3. Discussion

There are currently limited drugs and vaccines for the treatment or prevention of COVID-19. Various approaches have been utilized to combat SARS-CoV-2 infection, including inhibiting the viral replication of viral proteases and RNA-dependent RNA polymerase (RdRP) [27,28], as well as disrupting the interactions between the viral spike protein and host proteins ACE2 or TMPRSS2 [29,30].

**Figure 7:**
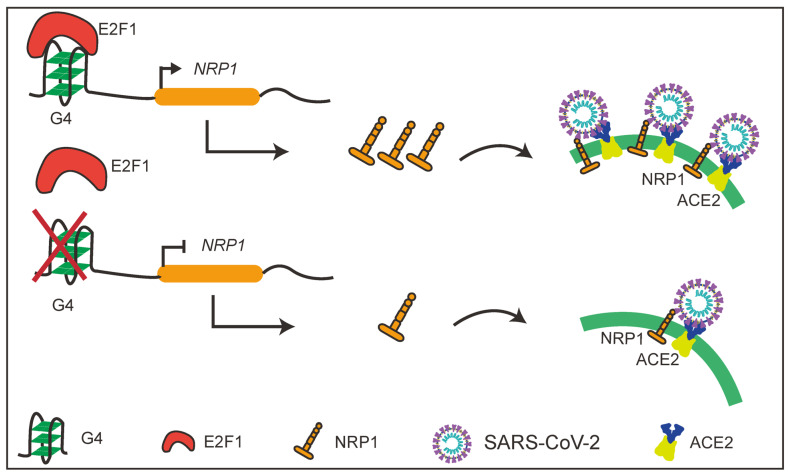
Schematic diagram of the study.

In this study, we discovered the presence of G4 structures in the promoter regions of several SARS-CoV-2 host factors with a focus on an important molecule NRP1. The presence of G4 structures in the *NRP1* promoter significantly promotes the expression levels of NRP1. Interestingly, we also found that disruption of the G4 structures in the promoter effectively blocked the entry of SARS-CoV-2 pseudovirus into cells. Furthermore, we identified the transcription factor E2F1 as the binding factor in the *NRP1* promoter (Figure 7). These findings highlight the importance of G4 structures in the promoter regions of host factors in SARS-CoV-2 entry and offer a promising mechanism-driven, broad-spectrum approach for the prevention and treatment of COVID-19.

Several studies have identified a substantial presence of RNA G-quadruplex (RG4) in SARS-CoV-2 [31,32]. These findings strongly suggest the involvement of RG4 in SARS-CoV-2 infection. Other studies highlight the significance RG4 in SARS-CoV-2 pathogenesis and provide an antiviral strategy for COVID-19 prevention [33]; they demonstrated that treatment with TMPyP4 significantly inhibits SARS-CoV-2 infection by cell-based assays. Moreover, the intranasal administration of TMPyP4 effectively suppresses SARS-CoV-2 replication and attenuates lung lesions with no observed toxicity in two animal models [11]. These findings emphasize RNA G-quadruplex as a promising therapeutic target for tackling COVID-19.

In this investigation, we elucidate the importance of G-quadruplex structures formed in the promoter regions of *NRP1* for SARS-CoV-2 entry. Numerous host factor promoters contain multiple G4-forming sequences known as PQSs, which adopt a characteristic G4 structure. Therefore, we extended the analysis of G4 to a common host factor, NRP1. We uncovered the biological, physiological, and pathological functions of G4 structures in gene regulation and their impact on SARS-CoV-2 infection. These findings shed light on a transcriptional regulatory mechanism underlying the pathogenesis of SARS-CoV-2, providing a promising avenue for the prevention and treatment of COVID-19.

G-quadruplex structures play crucial roles in various biological and physiological processes and have been implicated in the pathogenesis of several viral diseases [34]. In this study, we provide compelling evidence, for the first time, to characterize the existence and functional importance of G4 structures in host factors. We focus on NRP1, which is known to bind furin-cleaved substrates and significantly potentiates SARS-CoV-2 infection. Through bioinformatic analysis, we predicted the existence of multiple G4-forming sequences within the *NRP1* promoter. Subsequent biochemical and biophysical investigations confirmed that two of these sequences adopt canonical G4 structures. Notably, these canonical G4 structures significantly increase NRP1 expression level, thereby facilitating SARS-CoV-2 entry into cells. In severe COVID patients, the G4 sequences within the *NRP1* promoter region may tend to fold into G4 structures. The G4 structures on the *NRP1* promoter may be more stable and persist for a longer duration, potentially rendering individuals more susceptible to SARS-CoV-2 infection or experiencing frequent bouts of COVID. G4 on the *NRP1* promoter could be a potentially valuable biomarker for predicting COVID-19 complications, and targeting G4 on the *NRP1* promoter may yield favorable clinical outcomes in COVID-19 patients. It is worth noting that other host factor promoters also contain potential G4 structures, such as TMPRSS2, KREMEN1, DPP4, BSG, and AXL. The presence of G4 structures in these gene promoters is substantiated by G4 ChIP-seq data, pointing to a prevalent regulatory role of G4 structures in the promoters of host factors.

Despite the functional importance of NRP1 in COVID-19 pathogenesis, the molecular mechanisms governing its endogenous regulation remain largely elusive. In accordance with the findings presented in our study, which elucidates the role of G4 structures in promoting *NRP1* expression and facilitating SARS-CoV-2 infection, an scRNA-seq dataset also indicated elevated levels of *NRP1* expression in infected ciliated cells. Generally, G4 structures present in gene promoters exert their influence on gene expression by modulating the process of transcription, and transcriptional regulation is an essential biological process that enables the cell or an organism to respond to a range of intra- and extra-cellular signals. Here, we observed a substantial decrease in NRP1 protein levels when disrupting two G4 structures within the *NRP1* promoter, indicating a strong inhibitory impact on *NRP1* transcription. This implies that G4 structures within the promoter region, as well as other host factors, could potentially govern the transcriptional regulation. Considering the pivotal role of G4 structures in NRP1 expression and SARS-CoV-2 infection, it becomes crucial to identify the transcription factor (TF) binding to G4, as it is well-known that TF in promoters plays a role in the regulation of gene expression [35]. This study has pinpointed that the transcription factor E2F1, which is known for promoting gene transcription, binds to the G4 of *NRP1*. Of note, TMPyP4 is not a specific ligand that binds to the G4 structures in the *NRP1* promoter; its nonspecific binding might lead to low bioavailability or unpredictable side effects. Therefore, it is necessary to design specific molecular ligands that can bind to the G4 structures in the *NRP1* promoter to inhibit the binding of E2F1 and other transcription factors, thereby reducing NRP1 expression and inhibiting SARS-CoV-2 entry.

In conclusion, our findings, for the first time, showed that the G4 structure formed in the *NRP1* promoter region acts as a transcript activator. In addition, the G4 structure in the *NRP1* promoter also serves as a binding site for the transcription factor E2F1 to regulate the transcription of *NRP1*. G4s in the promoter of *NRP1* can significantly promote the expression level of NRP1, hereby facilitating NRP1-primed host cell entry of SARS-CoV-2 in vivo. All these results together provided new insights into the molecular mechanism of the G4 structure in the epigenetic regulation of *NRP1* gene transcription, and they showed the potential clinical application of G4-based drugs for COVID-19 prevention and treatment.

## 4. Materials and Methods

### 4.1. Cell Culture

A549, HepG2, and HEK293T cell lines were obtained from the American Type Culture Collection (ATCC, Manassas, VA, USA). The cells were cultured in Dulbecco’s Modified Eagle Medium (DMEM, Life Technologies, Carlsbad, CA, USA) supplemented with 10% fetal bovine serum (FBS, Invitrogen, Carlsbad, CA, USA) and 100 units/mL penicillin/streptomycin. The cultures were maintained at 37 °C in a humidified incubator with 5% carbon dioxide (CO_2_).

### 4.2. CD Spectroscopy

The oligonucleotide solutions in 20 mM lithium cacodylate (pH 7.0) with 100 mM KCl were annealed by heating at 95 °C for 5 min and then slowly cooled to room temperature. CD spectra were recorded on a Chirascan circular dichroism spectrophotometer (Applied Photophysics, Leatherhead, UK) using a 1 cm path length quartz cuvette at 25 °C with the following parameters: range 500−200 nm, bandwidth 1.0 nm, time per point 0.5 s, repeats 4. The wild-type and mutant sequences of two G4 motifs in the *NRP1* promoter region are shown in Table 1. Concentrations of oligodeoxyribonucleotides were in the range 0.5−1.0 μM. The absorbance change at each wavelength was adjusted to account for variations in temperature and plotted using the SigmaPlot 12.0 software.

### 4.3. Western Blot

The protein quantification was determined using a BCA assay. Subsequently, 40 μg of proteins from each sample was separated using SDS-PAGE and transferred onto a PVDF membrane. The membrane was then blocked with a 5% non-fat milk solution in TBST at room temperature for 1 h. Following the blocking step, the membrane was incubated overnight at 4 °C with primary antibodies specific to NRP1 (Abcam, Cambridge, UK) and ACTIN (Abcam, Cambridge, UK). Then, the membrane was incubated with appropriate secondary antibodies at room temperature for 1 h. Finally, the specific protein bands were visualized using an enhanced chemiluminescence detection kit (Thermo Fischer, Waltham, MA, USA; #32106).

### 4.4. RT–qPCR

Total RNA was isolated from cultured cells using TRIzol reagent (Invitrogen, Carlsbad, CA, USA) according to the manufacturer’s instructions. The reverse transcription of RNA to complementary DNA (cDNA) was performed using a reverse transcription kit (Takara, Dalian, China). Quantitative reverse transcriptase-polymerase chain reaction (qRT-PCR) was conducted on an ABI7900 real-time system (Applied Biosystems, Foster City, CA, USA) to analyze the expression levels of target genes. Gene-specific primers were utilized, and their details sequence can be found in Table 2. β-actin was used as an internal control for analysis, and the relative expression of mRNAs was determined using the comparative Ct method [36].

### 4.5. ChIP–qPCR

Cellular crosslinking was achieved by treating cells with 1% formaldehyde, which was followed by quenching with 0.125 M glycine. For ChIP, chromatin was isolated using the SimpleChIP Enzymatic Chromatin IP Kit (Cell Signaling Technology, Danvers, MA, USA), fragmented to a length of 200–300 base pairs through sonication, and subjected to immunoprecipitation using antibodies specifically targeting the BG4 or E2F1 antibody overnight. Subsequently, immunoprecipitated chromatin was precipitated and washed. The eluted chromatin was completely reversed by incubation overnight at 65 °C with proteinase K (Thermo Fisher Scientific, Waltham, MA, USA). The purification of the precipitated DNA was performed using the ChIP DNA Clean & Concentrator Kits (Zymo Research, Irvine, CA, USA). The precipitated DNA was coupled with PCR (ChIP-PCR) using the primers provided in Table 3.

### 4.6. Non-Denaturing Gel Electrophoresis

The oligonucleotides were resuspended in a solution containing 2 μM oligonucleotide concentration, 10 mM Tris-HCl, and 100 mM KCl. The mixtures were heated to 95 °C for 5 min and then cooled at room temperature overnight. Native polyacrylamide gels (15%) were prepared using 1× TBE buffer supplemented with 25 mM KCl. For electrophoresis, 10 μL of the oligonucleotide mixtures, respectively, were loaded onto the gel. The electrophoresis was performed at a voltage of 17 V/cm with a running time of 1 h at 4 °C. The gels were stained with SybrGold staining (Invitrogen, Carlsbad, CA) to confirm the bands.

### 4.7. Plasmid Construction and Viral Packaging

The full-length sequence of *NRP1* was synthesized by GenePharma (Shanghai, China) and subsequently cloned into the pcDNA3.1 vector. Additionally, we synthesized the wild and mutant type of the *NRP1* promoter (200 bp upstream of the TSS) and ligated them into pcDNA3.1 to replace the original promoter. The siRNAs targeting *E2F1* (sh-*E2F1*) and their negative control were synthesized by GenePharma (Shanghai, China) with the siRNA sequence provided in Table 4. Cells were transfected with above vectors or siRNA using Lipofectamine 2000 (Invitrogen, Carlsbad, CA, USA). A stable cell line, HEK293T-ACE2, overexpressing human ACE2, was generated by Obio Technology (Shanghai) Corp., Ltd. (Shanghai, China). Lentiviral infection was used in combination with puromycin (Cat# P9620, Sigma Aldrich, St. Louis, MO, USA) screening to construct the cell line. The cells were cultured in DMEM (Gibco, Grand Island, NY, USA) supplemented with 10% fetal bovine serum (FBS) (Gibco, Grand Island, NY, USA) and 1% penicillin/streptomycin (Gibco, Grand Island, NY, USA). The culture was maintained at 37 °C in a 5% CO_2_ atmosphere.

Obio Technology (Shanghai, China) Corp., Ltd. provided the SARS-CoV-2 spike pseudovirus, which can specifically infect cells expressing ACE2. The pseudovirus was derived from lentivirus and replaces the VSV-G envelope protein with the spike protein of SARS-CoV-2. Additionally, the pseudovirus carries the enhanced green fluorescent protein (EGFP) gene and luciferase reporter gene, facilitating detection in subsequent experiments.

### 4.8. ChIP-Seq Data and scRNA-Seq Data Processing

G4 ChIP-seq data were retrieved in the GEO database with accession number GSE133379; ChIP-seq reads were mapped to the human genome (UCSC hg38) using Bowtie version 1.1.2. Only reads that mapped uniquely with up to two mismatches within the 150 bp read length were retained. The resulting reads were then extended by 200 bp toward the center of the sequenced fragments and normalized to the total number of reads used for alignment, which was represented as reads per million (r.p.m.). The aligned reads in BAM format were further normalized to the total number of aligned reads and converted into bigwig files for visualization in the UCSC Genome Browser. Peak calling was performed using MACS2 (model-based analysis of ChIP-Seq) version 2.1.2 with default parameters [37].

The scRNA-seq files from infected human bronchial epithelial cells (HBECs) in air liquid interface (ALI) cultures were obtained via the GEO database (https://www.ncbi.nlm.nih.gov/geo/ (accessed on 10 March 2023)) with accession number GSE166766. We used the Seurat R package (version 3.0.2) to analyze the scRNA-seq data [38,39]. Standard downstream processing steps were applied, including filtering out genes detected in less than 3 cells and cells with fewer than 200 detected genes. We also limited the proportion of mitochondrial genes to less than 20%. Data normalization was performed using the LogNormalize method. To visualize and cluster the cell populations in a two-dimensional map, we applied the UMAP (Uniform Manifold Approximation and Projection) method after performing principal component analysis (PCA) [40]. Marker genes for each cluster were identified using the “FindAllMarkers” function with a filter criterion of absolute log2 fold change (FC) ≥ 1 and a minimum cell population fraction of 0.25 in either of the two populations. The expression pattern of each marker gene among clusters was visualized using the “DotPlot” function in Seurat.

### 4.9. DNA Pull-Down

The protein pull-down experiment was conducted using the BersinBio Bes5004 DNA pull-down kit (BersinBio, Guangzhou, China) following the manufacturer’s instructions. Biotinylated DNA oligonucleotide sequences containing Wt and Mut G4 structures were heated at 95 °C for 10 min in 50 mM Tris buffer at pH 7.5 with 100 mM KCl and then slowly cooled to room temperature over 4 h. We mixed 100 μL of pre-blocked streptavidin-coated magnetic polymer beads (Smart Life Sciences, Changzhou, China) with 150 nM of annealed double-stranded DNA in a 600 μL DNA binding buffer. Then, the streptavidin MagPoly beads with core promoter DNA were rotated with nuclear protein extract. Subsequently, the elution buffer was used to wash the bound proteins, which were then subjected to analysis using liquid chromatography-mass spectrometry (LC/MS) on a Q Exactive Mass Spectrometer (Thermo, Shanghai, China). The acquired mass spectrometric data were processed and analyzed using MaxQuant software (version 1.5.6.0) [41].

### 4.10. Dual-Luciferase Reporter Assay

The wild-type human *NRP1* promoter sequence and its mutants were synthesized by Sangon Biotech, Shanghai, China. They were then cloned into the psiCHECK-2 plasmid, replacing the promoter upstream of the hRluc reporter gene. The psiCHECK-2 plasmid contains both renilla luciferase (hRluc) and firefly luciferase (Fluc) genes. The constructed plasmids were used to transform DH5α competent cells. The transformed cells were harvested using the GeneJet Maxiprep kit (Thermo Fisher Scientific, Waltham, MA, USA) and verified by Sanger sequencing. The A549 and HEK293T cell lines were typically transfected with 1 μg of the plasmid DNA using Lipofectamine 2000 (Thermo Fisher Scientific, Carlsbad, CA, USA). After 36 h of transfection, the expression of the luciferase reporter gene was measured using the Dual-Glo kit from Promega (Madison, WI, USA), following the manufacturer’s instructions. The measurements were performed using a Veriscan Lux luminometer (Thermo Fisher Scientific, USA).

### 4.11. Statistical Analyses

An unpaired Student’s *t*-test was utilized for two independent group comparisons by GraphPad Prism 7 (GraphPad, La Jolla, CA, USA). All data were represented as mean ± SEM. Statistical significance is indicated as follows: * for *p* < 0.05 and ** for *p* < 0.01.

## Figures and Tables

**Table 1:** The 5′-3′ sequences of short single stranded sequences used in this study.

G4 Name	Sequence (5′-3′)
G1-Wt	GGGGAGAGGGGCGGGAGGAAGCGAGGGAAGGGC
G1-Mut	GAAGAGAGAAGCAAGAGGAAGCGAAAGAAGAAC
G2-Wt	TGGGGGGAGGCCGCAGGAGGGGAGGCGGGGGT
G2-Mut	TGAAGAGAGACCGCAAGAGAAGAGGCGAAAGT
*KIT*-Wt	AGGGAGGGCGCTGGGAGGAGGGG
*KIT*-Mut	AAAGAGAACGCTAAGAGGAGGAA

**Table 2:** Primer sequences used for reverse transcription-quantitative PCR.

Gene	Sequence (5′-3′)
*E2F1*	F: GGACCTGGAAACTGACCATCAG R: CAGTGAGGTCTCATAGCGTGAC
*NRP1*	F: AACAACGGCTCGGACTGGAAGA R: GGTAGATCCTGATGAATCGCGTG
*β-actin*	F: GTCATTCCAAATATGAGATGCGT R: GCTATCACCTCCCCTGTGTG

F, forward; R, reverse.

**Table 3:** Primer sequences used for ChIP-PCR.

Gene	Sequence (5′-3′)
*NRP1*	F: ACAGAGGAGTTTCACCAACTGC R: CCCAGTCTCCTGTCAGGCAATTA
*E2F1*	F: GCGGGAGACAGAGGAGTTTC R: TGTCAGGCAATTACAGGCGA

F, forward; R, reverse.

**Table 4:** siRNA sequence of genes used in this manuscript.

Gene	Sequence (5′-3′)
*E2F1*-siRNA	sense: GGACCTGGAAACTGACCATCAG
	antisense: CAGTGAGGTCTCATAGCGTGAC

## Data Availability

All data generated or analyzed during this study are included in the manuscript and/or the Supplementary Materials.

## Supplementary Materials

The supporting information can be downloaded at https://www.mdpi.com/article/10.3390/ijms25084422/s1.
