# Supplementary material for: DNA G-Quadruplex in NRP1 Promoter Facilitates SARS-CoV-2 Infection
Source: Int J Mol Sci. 2024 Apr 17;25(8):4422. doi: 10.3390/ijms25084422 (PMC11050221; doi:10.3390/ijms25084422)
Supplement: Supplementary file 1 [file ijms-25-04422-s001.zip › ijms-2955473-supplementary.pdf]

The top 30 proteins were screened out according to scores and densities in LC/MS/MS analysis

Gene names	Score	Intensity mut	Intensity wt1	Intensity wt2
YBX1	165.6	0	11492000	62983000
A2M	138.92	36784000	406750000	353820000
APOA1	112.05	46118000	148780000	279860000
SERPINA1	103.01	209450000	710530000	769690000
ITIH2	94.572	0	287610000	333450000
HNRNPM	85.737	45866000	142370000	6567500
IGHA1	83.861	221080000	17006000	53667000
HNRNPA2B1	81.919	265390000	190440000	51055000
DDX21	71.78	0	68853000	3432400
HNRNPA1	57.56	171170000	56472000	21731000
HEL-S-163pA	56.691	39847000	49346000	69009000
SERPINC1	45.66	93763000	38134000	46887000
NCL	43.1	24207000	33149000	12983000
E2F1	41.975	0	11128000	1713100
HBA1	38.953	79566000	22367000	30802000
CFB	38.904	0	335970000	610130000
CP	38.275	6540100	45273000	30197000
IGHM	35.366	17291000	546710000	529550000
DCD	31.359	103500000	41712000	23633000
SFPQ	30.333	32792000	42575000	12013000
HPX	29.586	5883000	141360000	418550000
SERPINA3	29.416	0	27603000	297020000
KNG1	28.138	401760000	6245700	14940000
ITIH4	26.585	0	161690000	222090000
SERPIND1	25.807	0	22125000	96624000
F2	25.492	3072800	52704000	39287000
PGLYRP2	23.916	0	37199000	32817000
PROS1	22.61	0	20412000	46547000
HNRNPD	21.974	11474000	15865000	9158400
APOB	21.144	0	73796000	67252000
